# Plasma procalcitonin and C-reactive protein concentrations in dogs with bacterial sepsis and non-infectious systemic inflammatory response syndrome

**DOI:** 10.3389/fvets.2025.1609020

**Published:** 2025-06-18

**Authors:** Johanna Rompf, Bérénice Lutz, Katja-Nicole Adamik, Eliane Marti, Jelena Mirkovitch, Laureen Michèle Peters, Jennifer Eiermann, Gertraud Schüpbach-Regula, Bianca Hettlich, Barbara Willi, Simone Schuller

**Affiliations:** ^1^Division of Small Animal Internal Medicine, Department of Clinical Veterinary Science, Vetsuisse Faculty, University of Bern, Bern, Switzerland; ^2^Small Animal Emergency and Critical Care Group, Department of Clinical Veterinary Science, Vetsuisse Faculty, University of Bern, Bern, Switzerland; ^3^Division of Neurological Sciences, Department of Clinical Research and Veterinary Public Health, Vetsuisse Faculty, University of Bern, Bern, Switzerland; ^4^Clinical Diagnostic Laboratory, Department of Clinical Veterinary Science, Vetsuisse Faculty, University of Bern, Bern, Switzerland; ^5^Veterinary Public Health Institute, Vetsuisse Faculty, University of Bern, Bern, Switzerland; ^6^Small Animal Surgery Division, Department of Clinical Veterinary Science, Vetsuisse Faculty, University of Bern, Bern, Switzerland; ^7^Clinic for Small Animal Internal Medicine, Vetsuisse Faculty, University of Zurich, Zürich, Switzerland

**Keywords:** canine, antimicrobial stewardship, biomarker, sepsis, SIRS

## Abstract

Procalcitonin is a well-established biomarker of bacterial infections in human medicine, used to guide initiation and duration of antimicrobial treatment. C-reactive protein (CRP) is a frequently used marker of inflammation in dogs, but is not specific for bacterial infection. The main objective of this study was to determine kinetics of plasma PCT (pPCT) and CRP in dogs with sepsis, non-infectious systemic inflammatory response syndrome (nSIRS) and healthy dogs. This prospective, observational study included 17 dogs with sepsis, 16 with nSIRS and 15 healthy dogs. Hematologic parameters, pPCT and CRP were assessed on days 1, 2 and 3 in healthy dogs and on days 1, 2, 3 and 4 in dogs with nSIRS or sepsis. The shortened Acute Patient Physiologic and Laboratory Evaluation (APPLE_fast_) score was calculated for dogs with sepsis and nSIRS. Plasma PCT was measured using a validated canine PCT ELISA. There was no significant difference in median pPCT between healthy dogs (110.3 pg/mL; IQR 74.7–138) and dogs with sepsis (81.6 pg/mL; IQR 50.1–157.1) or nSIRS (105.3 pg/mL; IQR 87.6–164.7). Prior antimicrobial treatment was not associated with a decrease in pPCT concentration in septic dogs. In the sepsis group, day 1 pPCT concentrations were significantly higher in non-survivors than in survivors (*p* < 0.05). In contrast, median CRP was above the reference range (<10.5 mg/L) in dogs with nSIRS (100.7 mg/L; IQR 67–141.9) or sepsis (131.9 mg/L; IQR 75.7–194.8) and significantly decreased within the first 4 days of successful antimicrobial treatment of sepsis. In conclusion, while plasma PCT showed some prognostic value, it was not a useful biomarker for assessing the efficacy of the chosen antimicrobial treatment in dogs with sepsis.

## 1 Introduction

Antimicrobials (AMs) are the mainstay of treatment of bacterial infections, but their use is unequivocally linked to the selection of resistant bacteria, threatening human and animal health (1, 2). The optimization of antimicrobial use is therefore one of the cornerstones in the fight against antimicrobial resistance. In order to ensure patient safety while avoiding over prescription, it is necessary to adapt antimicrobial treatments to the individual patient. To do this safely, biomarkers may aid clinicians to decide when to initiate and discontinue antimicrobial therapy (3).

In human medicine, procalcitonin (PCT) has been established over the past 20 years as a blood marker of bacterial infection. In health, PCT is synthetized by C-cells in the thyroid gland and released into the blood stream after proteolytic cleavage to calcitonin in response to increased plasma calcium concentrations (4). During bacterial infection, PCT expression by extra-thyroidal cells including neutrophils, monocytes and cells in liver, lung, spleen and kidneys is increased. In contrast, viral infections do not or only slightly increase PCT in humans (5). PCT has therefore been favored over C-reactive protein (CRP) as biomarker for the detection of bacterial infections. In human medicine, PCT-guided antimicrobial therapy has been shown to significantly shorten the duration of AM treatment without increase in morbidity or mortality for a wide range of infections including septic conditions like respiratory infections, pyelonephritis and surgical infections (6). However, more recent studies suggest that PCT may not be superior to CRP for monitoring of treatment response in humans with bacterial infections (7, 8).

In 2018 Goggs et al. published validation data of a commercial canine ELISA and initial analyses from several authors suggested that, despite some overlap, median PCT was significantly elevated in dogs with sepsis compared to healthy dogs (9–11). Also, PCT concentrations were found to be correlated with disease severity and outcome in septic canine patients (12). Furthermore, experimental data revealed that injection of lipopolysaccharide leads to a peak in PCT concentrations in dogs within 4 h of injection, returning to baseline at 48 h post injection, thus suggesting that PCT might be a useful biomarker for bacterial infection in dogs (11).

We hypothesized that (1) pPCT concentrations are significantly higher in dogs with bacterial sepsis than in dogs with non-infectious systemic inflammatory response syndrome (nSIRS) or healthy dogs, (2) pPCT decreases within the first 4 days of treatment in septic dogs with favorable clinical outcome, (3) initial pPCT concentrations are lower in dogs pretreated with antimicrobials compared to untreated dogs and (4) pPCT is superior to CRP, white blood cell (WBC) concentration and band neutrophil concentration to differentiate dogs with bacterial sepsis from dogs with nSIRS.

The aim of this study was to evaluate the kinetics of plasma PCT (pPCT) in dogs with bacterial sepsis and relevant control populations in order to estimate whether pPCT is a helpful tool to tailor antimicrobial therapy in dogs with sepsis.

## 2 Materials and methods

### 2.1 Study design

The study was designed as a prospective, observational trial. The study protocol was approved by the local ethics committee (BE 127/19). Written informed owner consent was obtained before enrolment of dogs in the study.

Healthy dogs and dogs meeting diagnostic criteria for nSIRS or sepsis presented to a small animal referral and teaching hospital between June 2020 and August 2021 were included.

All dogs were assessed at baseline (D1) via review of their medical history, physical examination including assessment of their mentation at presentation, hematology and blood biochemistry. In dogs with nSIRS and sepsis, blood lactate was also measured. Patient data of D1 (mentation, platelet concentration, albumin, glucose, lactate) then were used to calculate the shortened Acute Patient Physiologic and Laboratory Evaluation (APPLE_fast_) score retrospectively. Healthy controls were sampled at presentation (D1) and on two consecutive days (D2; D3) for practical reasons. Dogs with nSIRS or sepsis were sampled at admission (D1), D2, D3 and D4 for measurement of pPCT, plasma CRP and complete blood count (CBC). Other diagnostic tests and all treatment decisions were at the discretion of the attending clinician.

### 2.2 Dogs

*Healthy dogs* — Dogs were included if they had an uneventful medical history, had not received any medication in the previous 3 months except for preventative antiparasitic treatment, had a normal physical examination (eg. body temperature between 38.1°C and 39.2°C, heart rate between 60/min and 120/min depending on the size of the dog, respiratory rate <20/min), unremarkable CBC and plasma biochemistry profiles, including plasma CRP within the reference intervals (<10.5 mg/L).

*Dogs with non-infectious systemic inflammatory response syndrome* — A diagnosis of nSIRS was based on clinical criteria for nSIRS in dogs published by Hauptman et al. (13). Briefly, dogs were included if they fulfilled ≥ 2 of the following criteria at admission: body temperature < 38.1°C or > 39.2°C; heart rate > 120/min; respiratory rate > 20/min; white blood cell (WBC) count <6.0 × 10^9^/l (6 000/μl) or > 16.0 × 10^9^/l (16 000/μl), and percentage of band neutrophils >3% of the total WBC count, assessed by manual differential blood count. Additionally, diagnostic workup as described below revealed no evidence of bacterial infection; thus, bacterial infection was ruled out.

*Dogs with bacterial sepsis* — Bacterial sepsis was diagnosed in dogs with nSIRS and bacterial infection, which was confirmed by direct visualization of bacteria either on cytological or histopathological specimens, bacterial culture, or intraoperative evidence of a septic focus.

Samples were submitted to the on-campus bacteriology laboratory for aerobic and anaerobic culture as well as antimicrobial susceptibility testing.

### 2.3 Sample collection and handling

Venous blood samples were obtained by venipuncture of the cephalic or saphenous veins using a 21-gage needle or from an intravenous catheter. In the latter case, a purge sample of 0.3 mL of blood was discarded before taking the actual sample. Blood was collected into ethylenediaminetetraacetic acid (EDTA) (Sarstedt AG, Nümbrecht, Germany) and lithium heparin tubes (Monovette, Sarstedt AG Nümbrecht, Germany).

D1 samples for hematology and blood biochemistry analyses as well as D2, D3, D4 samples for hematology were immediately analyzed using an automated analyzer (ADVIA 2120i, Siemens Healthcare, Zürich, Switzerland; Cobas c501, Roche diagnostics, Basel, Switzerland). D1 blood gas analysis for assessment of the lactate concentration was also performed directly after blood collection using an automated analyzer (RAPIDPoint 500e Blutgasanalysesystem, Siemens Healthineers International AG, Zürich, Switzerland) directly after sampling.

Lithium heparin samples at D1, D2, D3 and D4 for measurement of pPCT and CRP were centrifuged within 1 h of sampling for 10 min at 20°C with a relative centrifugal force of 3,000 × g. Directly after that, plasma was separated and aliquoted into five micro tubes of 0.5 mL (Sarstedt AG, Nümbrecht, Germany), which were then frozen at −80°C until the day of analysis. Frozen storage time ranged between 1 and 9 months.

### 2.4 Measurement of plasma procalcitonin

Plasma PCT was measured batch wise using a previously extensively validated canine PCT ELISA (Biovendor, Asheville, USA), for measurement of pPCT in citrated and heparinized plasma samples (9, 14). To ensure that the assay produces reliable results in our hands, a dilutional study and investigation of intra- and inter-assay variability was conducted, showing acceptable dilutional linearity, as already shown in a previous study (15). All samples and standards were measured in duplicates. The assay was performed as done in a previous study using a 2-fold dilution of the samples (15) and following the manufacturers’ instructions. Samples were reanalyzed if the coefficient of variation (CV) between duplicates was above 15%. The upper limit of the assay was defined as the absorbance of the highest standard after having observed that linearity requirements were met. If the absorbance of a sample exceeded the absorbance of the highest standard, the sample was measured again at a higher dilution.

Intra-assay variability was calculated based on eight measurements of three samples (V1, V2, V3) as duplicates on one ELISA plate. Inter-assay variability was calculated based on measurements of three samples (V1, V2, V3) on all 12 ELISA plates showing intra- and inter-assay variabilities of 4 and 14%, respectively, (data not shown).

Samples from different days of the same dog were measured on the same plate except when further dilution was required.

### 2.5 Measurement of C-reactive protein

Plasma CRP was measured batch wise using an automated analyzer Cobas c501 (Roche Diagnostics, Basel, Switzerland) with a previously validated CRP assay (Gentian Canine CRP; Scil Animal Care company, Viernheim, Germany) (16). The lower limit of quantification was set at 6.4 mg/L by the manufacturer; the upper limit of the reference interval was 10.5 mg/L.

### 2.6 Hematology

White blood cell concentrations were obtained using an automated analyzer (ADVIA 2120i, Siemens Healthcare, Zürich, Switzerland) with the following veterinary software: ADVIA multispecies software, version 6.3.2. A manual differential blood count based on 200 leucocytes was performed by experienced laboratory technicians to calculate absolute segmented and band neutrophil concentrations.

### 2.7 Measurement of blood lactate

Blood lactate concentrations were measured using an automated analyzer (RAPIDPoint 500e Blutgasanalysesystem, Siemens Healthineers International AG, Zürich, Switzerland).

### 2.8 Statistical analysis

Statistical analysis was performed using a commercial statistical software package (NCSS 2020, LLC. Kaysville, Utah, USA).

Statistical power and sample size analysis were performed using the following software: Sergeant, ESG, 2018. Epitools Epidemiological Calculators; Ausvet^1^ based on pPCT concentrations of healthy dogs in the current cohort and previously reported concentrations in septic dogs (9). This was decided in order to get the best possible results, based on the fact that pPCT of the healthy dogs in the present study were higher than in previous studies. The mean of 110 pg/mL and variance of 1,600 pg/mL of the cohort of healthy dogs from the present study was used to detect a difference of 40 pg/mL in mean PCT between control and septic dogs with a power of 80% and a significance level of 0.05. Power analysis indicated that at least 28 subjects per group were needed.

Patient characteristics including sex, neuter status, age, body weight and baseline blood variables were analyzed using descriptive statistics. Shapiro Wilk testing indicated non-normal distribution for several continuous variables, which therefore were reported as median and interquartile range (IQR).

Day-to-day variability and inter-individual variability of pPCT were assessed in the group of healthy dogs by calculation of CVs (%) via the following formula: Standard deviation/Mean*100%.

Differences in concentrations of pPCT, markers of inflammation including WBC concentration, band neutrophil concentration, and CRP were compared between disease groups and between survivors and non-survivors in nSIRS and sepsis groups using the Friedman’s Rank Test with Bonferoni’s *post-hoc* adjustment for multiple comparisons, which resulted in a corrected significance level of *p* < 0.008.

Associations between D1 pPCT concentrations and antimicrobial pretreatment or APPLE_fast_ score were assessed using Kruskal Wallis Test.

Because of the non-normal distribution of the data, the correlation of pPCT and CRP, and the associations of pPCT with clinical and laboratory variables were assessed using the Spearman rank correlation test. R values between +/− 0.5 and +/− 1 were defined as a strong correlation, values between +/− 0.3 and +/− 0.49 were defined as a moderate correlation and values below +/− 0.29 were defined as a weak correlation.

Statistical significance was set at *p* < 0.05.

## 3 Results

### 3.1 Study population

A total of 48 dogs were enrolled in the study: 17 dogs were diagnosed with bacterial sepsis, 16 with nSIRS, and 15 dogs were healthy. Population characteristics, baseline laboratory parameters, duration of hospitalization and outcome of dogs included in the study are summarized in Table 1. A positive outcome was defined as alive at discharge. Twelve dogs were crossbreds and 36 were purebreds belonging to 25 different breeds, the most common breeds being Border Collie (*n* = 3), German Shepherd dog (*n* = 3) and Labrador Retriever (*n* = 3). Of the 15 healthy dogs, 5 belonged to a cohort of military dogs. Of these 5 dogs, 2 were Malinois Shepherds, 2 were German Shepherds and 1 was a Dutch Shepherd. There were no significant differences between groups regarding age, neuter status, and body weight.

**Table 1:** Patient characteristics of healthy dogs and dogs with non-infectious systemic inflammatory response syndrome or sepsis.

Variable	Healthy		nSIRS		Sepsis	
	*n*	Median (IQR)	*n*	Median (IQR)	*n*	Median (IQR)
Age (y)	15	4.5 (2–7.8)	16	5 (1.8–9.3)	17	8 (5–11)
Body weight (kg)	15	24.8 (23.1–29.7)	16	30 (19.2–32.1)	17	33 (18.6–35.6)
Body Condition score	15	6 (4–6)	16	5.5 (2–7)	17	5 (3–7)
Sex	15		16		17	
Male (*n*, intact/ neutered)		6 (2/4)		13 (7/6)		8 (2/6)
Female (*n*, intact/ neutered)		9 (1/8)		3 (0/3)		9 (2/7)
APPLE_FAST_ score		NA	16	18.5 (17–21)	17	21 (19–22)
Neutrophil count (10^9^/l)	15	8.25 (6.9–10.1)	16	20.8 (12.7–24.8) **	17	15.6 (8.27–21.05)
Band neutrophils (%)	15	0 (0–0.1)	16	0.9 (0.5–1.7) **	17	1.67 (0.24–5.95) **
Albumin (g/l)	15	36.4 (35.4–37.3)	16	27.2 (24.2–33.2) **	17	21.5 (17.1–24.4) **#
CRP (mg/l)	15	<6.4	16	100.7 (67.0–141.9) **	17	131.9 (75.7–194.8)**
PCT (pg/ml)	15	110.3 (74.7–138.0)	16	105.3 (87.6–164.7)	17	81.6 (50.1–157.1)
Duration of hospital stay (d)		NA	16	3 (2–6)	17	6 (4–8)
Outcome						
Alive to discharge, *n* (%)		NA	15	15/16 (93.75)	12	12/17 (71)
Euthanized/ died, *n* (%)		NA	1	1/0 (6.25)	5	3/2 (29)

nSIRS, Non-infectious systemic inflammatory response syndrome; IQR, Interquartile range; PCT, procalcitonin; CRP, C-reactive protein; NA, not applicable; APPLE, Acute Patient Physiologic and Laboratory Evaluation.

** *p* < 0.001 vs healthy control; * *p* < 0.05 vs healthy control. # *p* < 0.05 vs SIRS.

All non-surviving dogs in the study died or were euthanized because of intractable disease with poor prognosis. No dog was euthanized because of financial reasons. Non-surviving dogs were diagnosed with the following: septic peritonitis (*n* = 3), pneumonia (*n* = 2), pyometra (*n* = 1), intestinal sarcoma (*n* = 1).

Median APPLE_fast_ score was 21 (IQR 17–22); in septic dogs it was 21 (IQR 19–22) and in dogs with nSIRS 18.5 (IQR 17–21), the difference was not significant. Dogs with nSIRS and sepsis had significantly lower concentrations of plasma albumin compared to healthy dogs on D1.

In septic dogs, the most common sources of sepsis were septic peritonitis (*n* = 9) and pyometra (*n* = 3) and one of each of the following: pyothorax, pyelonephritis, abscess formation at the level of the neck. In two dogs in the sepsis group, bacterial pneumonia was strongly suspected but could not be confirmed definitively. As those dogs were not sufficiently stable to undergo bronchoalveolar lavage, diagnosis was based on clinical and radiographic signs as well as response to antimicrobial therapy.

Samples analyzed from dogs in the sepsis group included abdominal fluid, pleural effusion, abscess material, and tissue biopsies, depending on the clinical presentation. Isolated bacteria included *Staphylococcus pseudintermedius* (*n* = 1), *Streptococcus equi* subsp. Zooepidemicus (*n* = 1), *Streptococcus anginosus* (*n* = 1), *Klebsiella pneumoniae* (*n* = 2), *Escherichia coli* (*n* = 2), *Enterobacter cloacae* (*n* = 1), and *Enterococcus faecium* (*n* = 1).

Half of the dogs in the sepsis group (10/17; 58.8%) were treated before hospital admission with one or more of the following antimicrobials: ampicillin-sulbactam (*n* = 2), amoxicillin (*n* = 3), cefovecin (*n* = 1), enrofloxacin (*n* = 3), metronidazole (*n* = 4), marbofloxacin (*n* = 2), unknown antimicrobial (*n* = 1).

Dogs with nSIRS were diagnosed with steroid-responsive meningitis-arteritis (*n* = 4), heat stroke (*n* = 2), gastric dilation without torsion (*n* = 2) and one of each of the following: suspected acute toxic liver insult, intestinal sarcoma, Shar-Pei Fever, acute neutrophilic splenitis, primary immune-mediated polyarthritis, acute severe gastroenteritis, acute pancreatitis, gastric dilation volvulus.

Prior to admission, one third of the dogs in the nSIRS group (5/16; 31.3%) had received one or more antimicrobials, including amoxicillin-clavulanic acid (*n* = 1), enrofloxacin (*n* = 1), and an unknown antimicrobial (*n* = 4). The remaining dogs had not received antimicrobial treatment before presentation.

### 3.2 Baseline procalcitonin and C-reactive protein plasma concentrations

**Figure 1:**
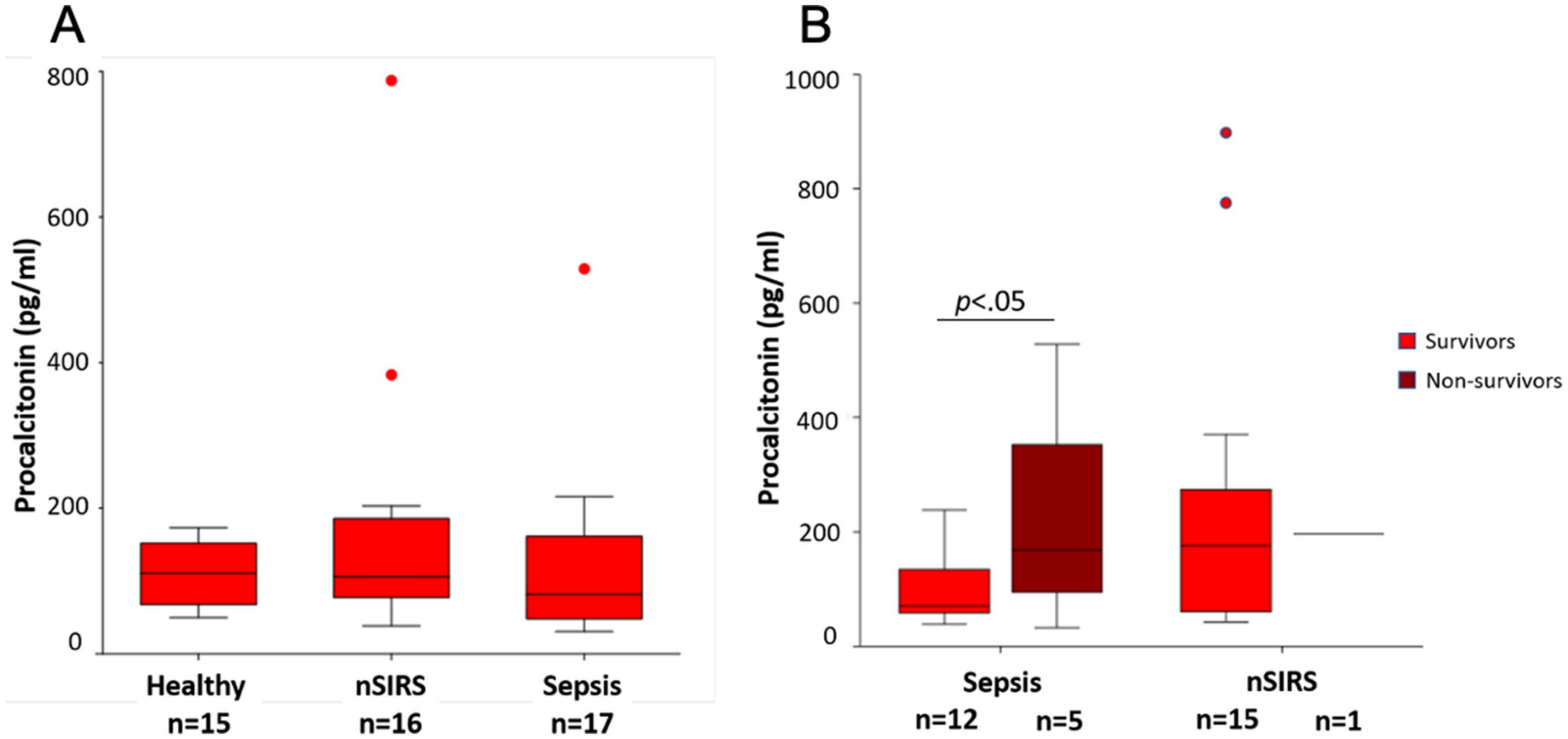
Comparison of median procalcitonin concentrations at presentation **(A)** in healthy dogs, dogs with non-infectious systemic inflammatory response syndrome (nSIRS) or sepsis in comparison; **(B)** in survivors versus non-survivors. The central lines in the boxes represent the median values, and the top and bottom of the boxes represent the 75th and 25th percentiles, respectively. The dots represent outliers.

At presentation, median pPCT concentrations did not significantly differ between dogs with sepsis, nSIRS and healthy dogs (Figure 1A). Median pPCT at baseline was significantly higher in non-survivors (*p* < 0.05) than survivors in the sepsis group (Figure 1B). This is based on *n* = 5 non-survivors. In the nSIRS group only 1/16 dogs died, therefore the statistical comparison between survivors and non-survivors was not performed.

**Figure 2:**
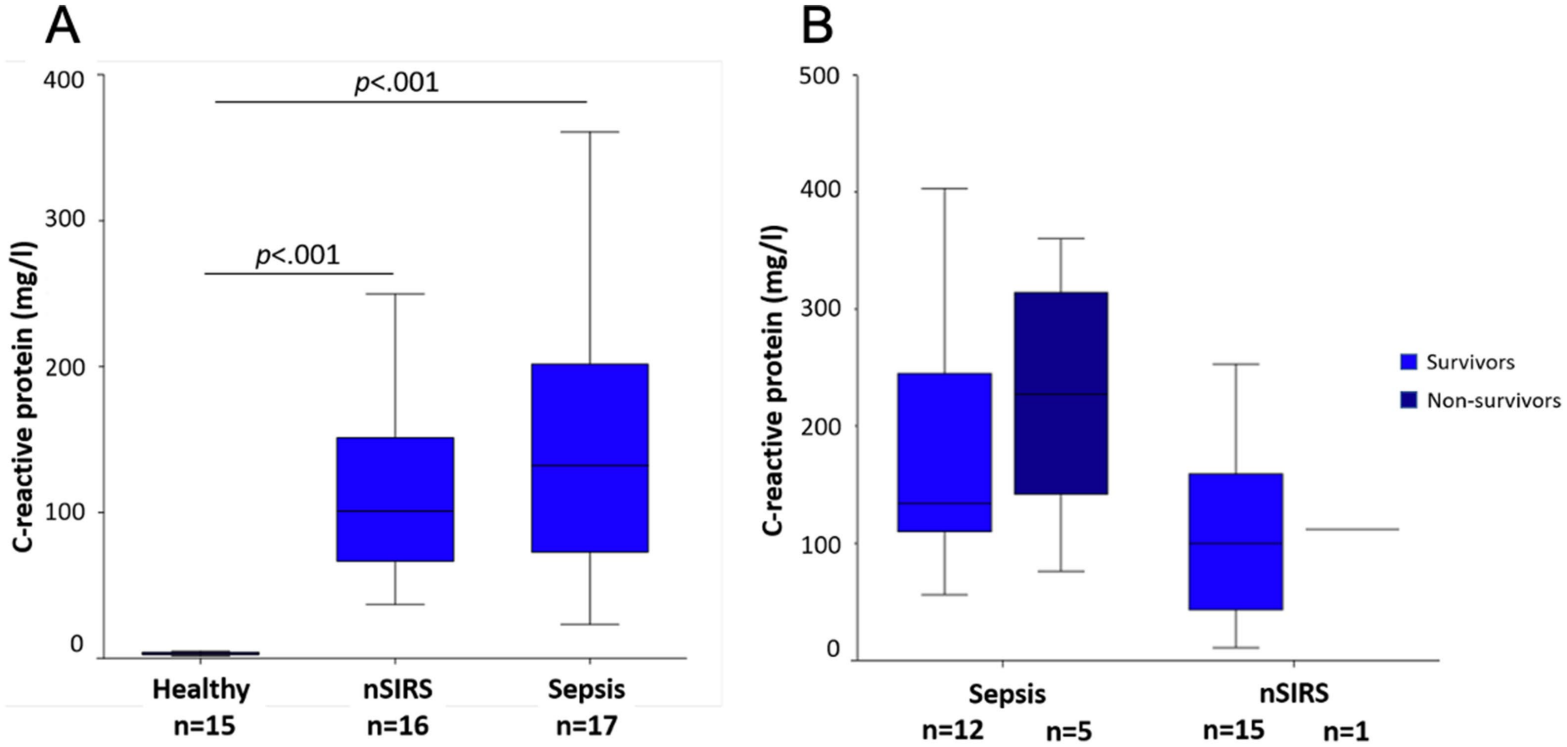
Comparison of median C-reactive protein concentrations at presentation **(A)** in healthy dogs, dogs with non-infectious systemic inflammatory response syndrome (nSIRS) or sepsis in comparison; **(B)** in survivors versus non-survivors. The central lines in the boxes represent the median values, and the top and bottom of the boxes represent the 75th and 25th percentiles, respectively. The dots represent outliers.

Plasma CRP concentrations at presentation were above the upper reference limit of the laboratory (10.5 mg/L) in all dogs with nSIRS or sepsis. Median CRP concentrations were not significantly different between sepsis and nSIRS groups (Figure 2A). There was no significant difference in median baseline CRP concentrations between survivors and non-survivors in the sepsis group (Figure 2B).

### 3.3 Kinetics of procalcitonin and C-reactive protein

**Figure 3:**
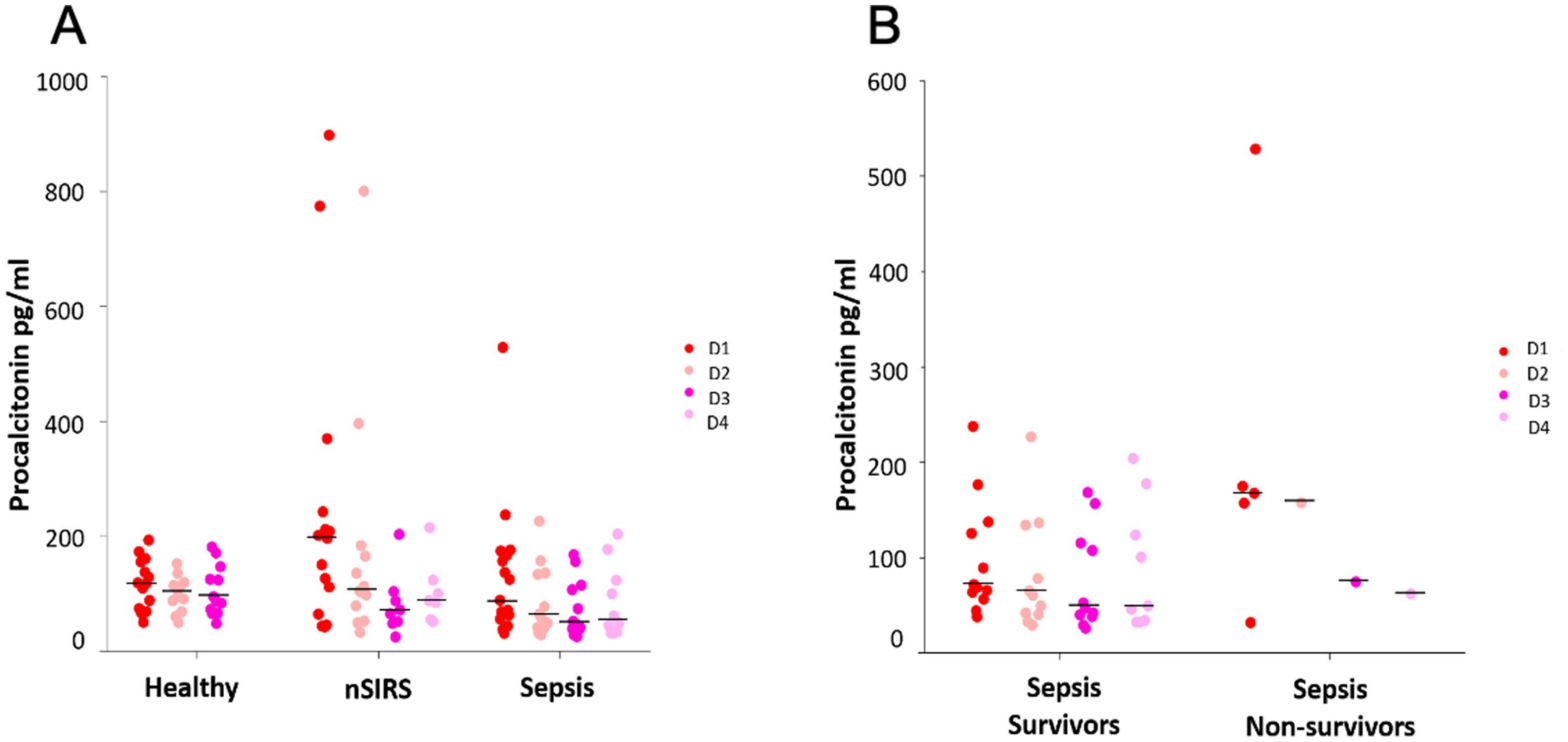
Kinetics of procalcitonin over 3 and 4 consecutive days in **(A)** healthy dogs versus dogs with non-infectious systemic inflammatory response syndrome (nSIRS) and dogs with sepsis, **(B)** survivors versus non-survivors in dogs with sepsis. The dots represent procalcitonin concentrations of each individual dog. The black lines represent medians. The non-survivors’ section of panel **B** shows values of the same dog on D2, D3, D4.

Comparison of median pPCT concentrations between D1, D2, D3 and D4 showed no significant differences between sampling time points in neither healthy, sepsis nor nSIRS groups (Figure 3A). Intra- and interindividual variabilities (CV_I_ and CV_G_, respectively) were markedly higher in the sepsis (CV_I_ 31%/ CV_G_ 75%) and nSIRS groups (CV_I_ 31%/ CV_G_ 85%) compared to the healthy group (CV_I_ 15%/ CV_G_ 36%), with individual animals showing markedly increased pPCT on one (*n* = 2) or several (*n* = 1) time points. Two dogs in the nSIRS group with high pPCT (>400 pg/mL) were diagnosed with heat stroke and steroid responsive meningitis arteritis. In the dog in the sepsis group with a PCT of 529 pg/mL bacterial pneumonia was suspected; the dog died shortly after sampling.

Comparison of pPCT kinetics between sepsis survivors and non survivors showed low and stable median pPCT, whereas in the one non-surviving dog with sepsis, who survived beyond day 1, pPCT initially increased and then decreased over time (Figure 3B).

**Figure 4:**
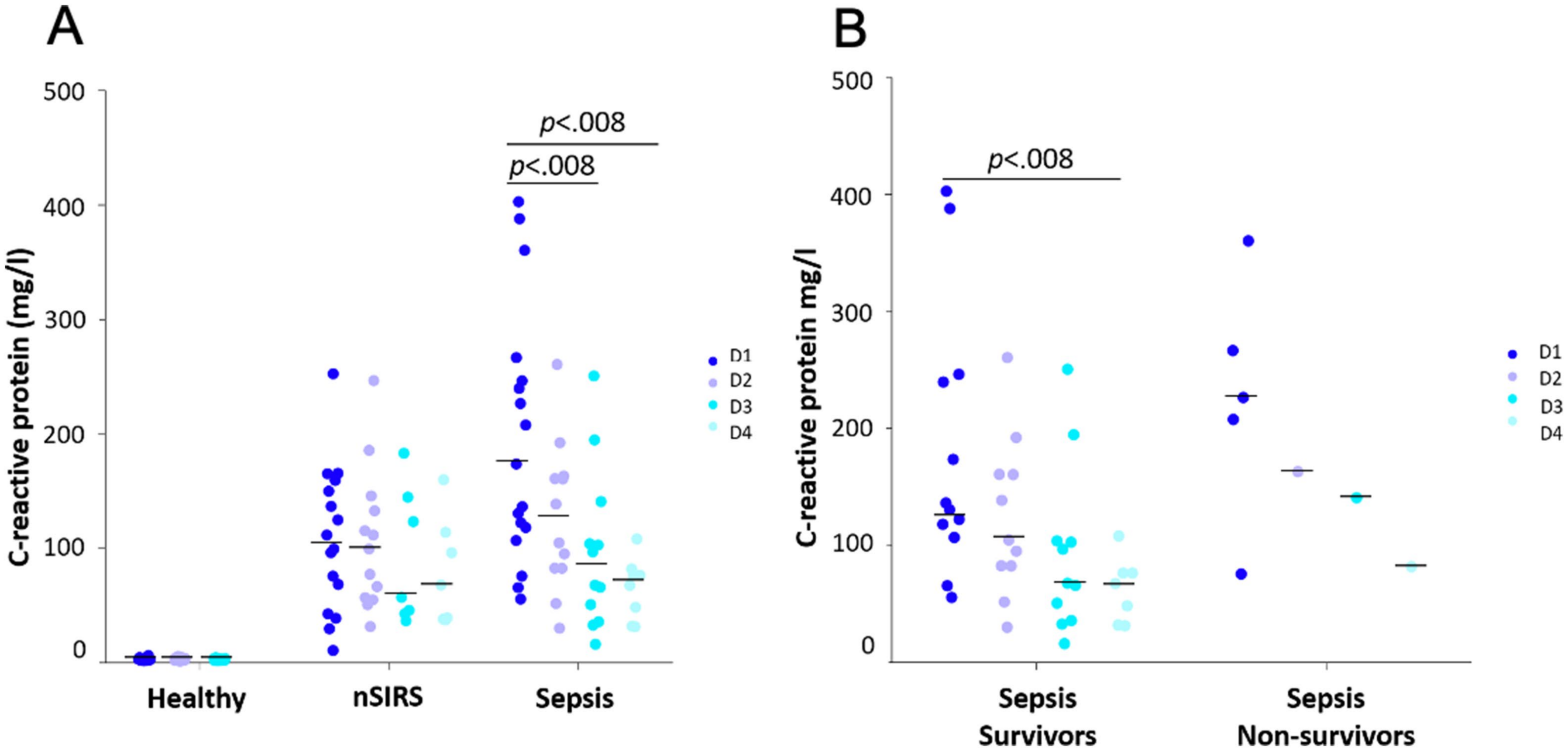
Kinetics of C-reactive protein over 3 and 4 consecutive days in **(A)** healthy dogs versus dogs with non-infectious systemic inflammatory response syndrome (nSIRS) and dogs with sepsis, **(B)** survivors versus non-survivors in dogs with sepsis. The dots represent C-reactive protein concentrations of each individual dog. The black lines represent medians. The non-survivors’ section of panel **B** shows values of the same dog on D2, D3, D4.

In dogs with nSIRS and sepsis, median CRP concentration progressively decreased over time. In dogs with sepsis, median CRP concentrations decreased significantly between day 1 and day 3 as well as day 1 and day 4 (D1/D3: *p* < 0.008; D1/D4: *p* < 0.008) (Figure 4A). In survivors, CRP concentrations decreased significantly between day 1 and day 4 (*p* < 0.008). Interpretation of kinetics in non-survivors is limited, as only one dog survived beyond the first day of sampling (Figure 4B).

### 3.4 Correlation analysis

**Figure 5:**
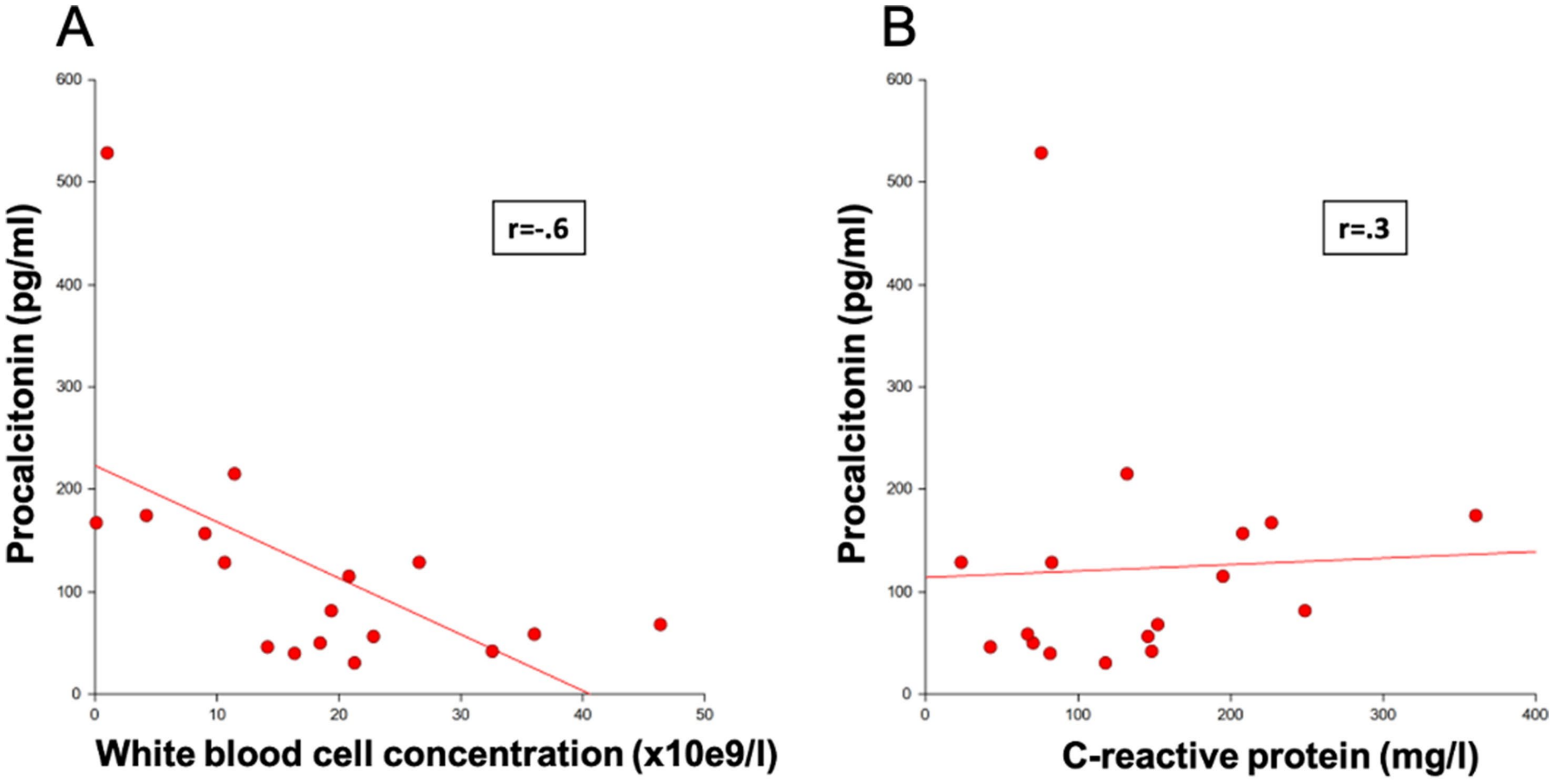
Correlation of procalcitonin concentrations in septic dogs with **(A)** white blood cell concentration and **(B)** C-reactive protein.

In septic dogs, median pPCT concentration was significantly and strongly negatively correlated with WBC concentration (*r* = −0.6; *p* < 0.05) (Figure 5A). No statistically significant correlation was found between median pPCT and CRP concentrations (Figure 5B), band neutrophil concentrations or albumin.

In dogs with nSIRS, no statistically significant correlation was found between median concentrations of pPCT and CRP, WBC, band neutrophil concentration or albumin.

There was no association between pre-treatment with antimicrobials and median pPCT concentrations in septic dogs. No statistically significant correlation was found between the APPLE_fast_ score and D1 pPCT and CRP concentrations in septic dogs.

## 4 Discussion

In this cohort of dogs, median pPCT concentrations were not significantly different between healthy dogs, dogs with nSIRS and dogs with bacterial sepsis at admission or at any of the other sampling days. Median pPCT at admission was significantly higher in non-surviving dogs with bacterial sepsis, compared to survivors. There was no correlation in pPCT between septic dogs pre-treated with antimicrobials and non-pretreated dogs. Together, these results indicate that pPCT might be of value as a prognostic marker, but it is not a suitable biomarker to identify dogs with sepsis or to guide antimicrobial therapy.

In contrast, median plasma CRP concentrations were markedly above the reference range in dogs with nSIRS and sepsis at admission. Furthermore, median CRP concentrations significantly decreased over the first 4 days of treatment in septic dogs with a favorable outcome. These results suggest that CRP is likely a more useful biomarker than pPCT to guide antimicrobial therapy in dogs with sepsis.

This study was based on results from a canine sepsis model showing pPCT peak concentrations 4 h after injection of lipopolysaccharide with a normalization within 48 h, indicating that pPCT is overexpressed in dogs in response to gram-negative bacterial infection, similar to what has been described in humans (5, 11). Furthermore, early clinical studies by the group of Goggs et al. indicated a significant elevation in median pPCT in dogs with bacterial sepsis compared to healthy controls (9, 12, 17). In later studies by the same group, however, pPCT was reported to be low in dogs with pneumonia, septic peritonitis or pyometra (18). The results of the present study unfortunately support those results.

The described discrepancies between older and more recent studies might be in part due to differences in study populations. In the study of Troia et al. (12), a large number of dogs with parvovirosis (15/53; 28%) were included in the sepsis group. In the present study, over half of the septic dogs (9/17; 53%) were diagnosed with septic peritonitis and dogs with parvovirosis were excluded, as this represents a very specific condition which is hardly comparable to other causes of sepsis. Our population of dogs therefore is closer to the one of the recent study by Goggs et al. (18), reporting no significant elevation in pPCT in dogs with septic peritonitis compared to an earlier defined reference range in healthy dogs.

A second possible explanation for the discrepancies between pPCT concentrations in older and more recent studies may be differences in disease severity. In the study of Troia et al. (12), pPCT was significantly higher in dogs with septic shock compared to dogs with sepsis without cardiovascular compromise. In the present study, dogs in the sepsis group were not further subclassified.

Another study by Matur et al. (10) suggests a possible correlation between disease severity and pPCT concentrations in their cohort of dogs. Disease severity in the present study was assessed by calculation of the APPLE_fast_ score, which resulted in a median value of 21 (19–22) in the sepsis group. Septic dogs in the earlier mentioned study by Troia et al. had an overall higher APPLE_fast_ score of 24 (14–33). Also the mortality of 18% in our study was far below the mortalities of septic patients reported in the earlier studies by Goggs et al. and Troia et al. of 30 and 28%, respectively (9, 12).

On the other hand, in the newer study of the same group, which demonstrated continuously low median pPCT concentrations in septic patients, an APPLE_fast_ score of 20 (14–26) and a mortality of only 8% were calculated (5). It is possible that patients in more recent studies including our present study were less seriously affected, as reflected by lower APPLE_fast_ scores and mortality rates, and therefore showed lower pPCT concentrations compared to older studies.

As stated before, in the experimental study by Easley et al. (11) pPCT peak concentrations were observed at about 4 h after injection of lipopolysaccharide. As our hospital is a referral center, we might have missed the peak due to too late presentation of affected dogs.

Finally, while pPCT concentrations of septic dogs in this study were comparable to previous studies, pPCT in our cohort of healthy dogs was markedly higher than previously reported, thus reducing the discriminatory power of pPCT further (9). Although the same assay has been used across studies, with almost no modifications, differences in methodology causing the discrepancies in pPCT concentrations in healthy dogs cannot be excluded.

Furthermore, the reference range defined by Goggs et al. in 2018 is based on citrated plasma samples (9), while we used heparinized plasma. The kit is validated by the manufacturer for use in serum and urine, the assay was later extensively validated for use in citrated and heparinized plasma (9, 14). While preliminary results indicated that the assay performs well in citrated, heparinized plasma and serum and gives comparable results between different sampling types (15), we cannot exclude that the higher measurements in healthy dogs are due to it being measured in heparinized rather than citrated plasma (19).

We found a strong negative correlation of WBC count and pPCT concentration on the day of admission in septic dogs. This might indicate pPCT to be an early marker of disease severity in canine sepsis, as in human medicine it has been proven that leucopenia is correlated with increased risk of mortality in sepsis (20). Troia et al. (12) describes a similar result; however, in their cohort most septic dogs had parvovirus enteritis, which might have influenced their overall leucocyte counts.

In contrast to pPCT, CRP was significantly above the reference limit (< 10.5 mg/L) in dogs with bacterial sepsis and nSIRS. However, there was no significant difference in CRP concentrations between dogs with sepsis and those with nSIRS. CRP therefore is not a useful marker to discriminate between sepsis and nSIRS, but reflects systemic inflammation in dogs, which is in agreement with a previous study (21).

Furthermore, CRP concentrations decreased significantly over the first 4 days of treatment in successfully treated septic dogs, mirroring disease resolution in these dogs. This is in accordance with other studies suggesting that CRP is a useful marker to monitor animals with bacterial infections and to guide antimicrobial treatment (18, 21).

In humans, several studies equally revealed that CRP kinetics could be used to guide treatment decisions in septic patients (22, 23).

Another factor for CRP being superior to pPCT is the easy availability of CRP by measurement in automated analyzers in almost every laboratory, in both veterinary and human medicine (24).

This prospective study had several limitations, most importantly the small size of population in all of our study groups, which results in a low power of the study. Power calculation prior to the study indicated that we would need at least 28 dogs per group in order to make a serious statement on significant differences between groups. However, when the first batches of samples were measured there was detected no difference at all between study groups and additionally, it was quite challenging to definitively rule out bacterial sepsis wherefore a large number of recruited patients had to be excluded after initial inclusion. For those reasons, we decided to stop recruitment of patients before reaching the number of 28 dogs per study group.

Secondly, the APPLE_FAST_ score was determined retrospectively, which might have limited the accurate assessment of the mentation score. Thirdly, we used the SIRS criteria by Hauptman et al. from 1997. By now those criteria have been replaced by newer ones. We still decided to use the criteria by Hauptman et al. to have better comparability with previous studies using the same criteria. A fourth limitation is the few number of non-survivors in the nSIRS group, which made relevant statistical analysis impossible. Also, only one non-surviving dog in the sepsis group had measurements on D2-D4, wherefore a statement on kinetics in non-surviving septic dogs cannot be made.

Additionally, dogs were classified to the best of our knowledge into the different groups based on available clinical, laboratory, and imaging data; however, bacterial translocation in dogs in the nSIRS group or underlying bacterial infection – particularly in patients pretreated with antibiotics – cannot be definitely excluded.

A last limitation is the inclusion of a cohort of military dogs in the group of healthy dogs, which have a different lifestyle than pet dogs and were of relatively similar breeds (mainly German Shepherds and Malinois).

In summary, the concentrations of pPCT in dogs with sepsis did not differ from those in dogs with nSIRS or healthy dogs, calling into question its utility as a diagnostic marker for bacterial sepsis or to guide antimicrobial therapy. Nevertheless, baseline levels of pPCT were significantly higher in non-survivors than in survivors, which may give PCT a role as a prognostic marker. In contrast, the kinetics of plasma CRP followed closely the clinical course of disease in septic dogs, as concentrations were significantly above the reference range in both sepsis and nSIRS and decreased significantly over the first 4 treatment days in dogs with sepsis. At present, CRP therefore remains the most useful and widely available marker to potentially guide AM treatment decisions in dogs.

## Data Availability

The original contributions presented in the study are included in the article/supplementary material, further inquiries can be directed to the corresponding author.

## Footnotes

^1^ http://epitools.ausvet.com.au
